# Comparative Genomic Analysis of *Bacillus velezensis* BRI3 Reveals Genes Potentially Associated with Efficient Antagonism of *Sclerotinia sclerotiorum* (Lib.) de Bary

**DOI:** 10.3390/genes15121588

**Published:** 2024-12-11

**Authors:** Yaoyao Liu, Changyan Yin, Min Zhu, Yuhua Zhan, Min Lin, Yongliang Yan

**Affiliations:** National Key Laboratory of Agricultural Microbiology, Biotechnology Research Institute, Chinese Academy of Agricultural Sciences, Beijing 100081, China

**Keywords:** *Bacillus velezensis*, fungal antagonistic, genome sequencing, comparative genomics

## Abstract

Background/Objectives: *Bacillus velezensis* has recently received increased attention as a potential biological agent because of its broad-spectrum antagonistic capacity against harmful bacteria and fungi. This study aims to thoroughly analyze the genomic characteristics of *B. velezensis* BRI3, thereby providing theoretical groundwork for the agronomic utilization of this strain. Methods: In this work, we evaluated the beneficial traits of the newly isolated strain *B. velezensis* BRI3 via in vitro experiments, whole-genome sequencing, functional annotation, and comparative genomic analysis. Results: *B. velezensis* BRI3 exhibits broad-spectrum antifungal activity against various soilborne pathogens, displays inhibitory effects comparable to those of the type strain FZB42, and exhibits particularly effective antagonism against *Sclerotinia sclerotiorum* (Lib.) de Bary. Whole-genome sequencing and assembly revealed that the genome of BRI3 contains one chromosome and two plasmids, which carry a large amount of genetic information. Moreover, 13 biosynthetic gene clusters (BGCs) involved in the biosynthesis of secondary metabolites were predicted within the BRI3 genome. Among these, two unique BGCs (cluster 11 and cluster 13), which were not previously reported in the genomes of other strains and could potentially encode novel metabolic products, were identified. The results of the comparative genomic analysis demonstrated the genomic structural conservation and genetic homogeneity of BRI3. Conclusions: The unique characteristics and genomic data provide insights into the potential application of BRI3 as a biocontrol and probiotic agent.

## 1. Introduction

Plant diseases pose a considerable threat to global crop yield and security [1]. To satisfy the escalating food demand of a burgeoning population, there has been a marked increase in the application of agricultural fungicides. In the past few decades, agricultural fungicides have undeniably played a pivotal role in advancing agricultural productivity; however, related concerns are concomitantly increasing. These include concerns regarding soil environment pollution, water pollution, air pollution, food safety threats, and the development of antibiotic resistance [2,3]. As a result, the application of biological control methods rather than fungicides has recently emerged as a more ecofriendly long-term strategy for managing plant diseases. Plant growth-promoting rhizobacteria (PGPR), such as *Bacillus* spp., play crucial roles in ecofriendly and sustainable agricultural practices [4]. A new species of *Bacillus* spp., designated *B. velezensis*, was initially identified in 2005 from samples of saltwater collected from the Vélez River in southern Spain [5]. Following the initial discovery, comparative genomic investigations in 2015 and 2016 revealed homologous evolutionary proximity of *Bacillus amyloliquefaciens* subsp. *plantarum* and *Bacillus methylotrophicus* with *B. velezensis* [6,7]. There has been a marked increase in research on the application of *B. velezensis* in biocontrol strategies, with a particular emphasis on the type strain *B. velezensis* FZB42, which has been extensively utilized across diverse research domains. *B. velezensis* FZB42, initially recognized as the type strain of *B. amyloliquefaciens* subsp. *plantarum*, has been the subject of extensive research. Following the taxonomic revisions proposed by Dunlap, the strain was redesignated *B. velezensis* FZB42 [6,7]. FZB42 and its related strains have been shown to exhibit broad-spectrum antagonistic effects against various plant pathogenic fungi, with confirmed efficacy in suppressing pathogens associated with cotton, strawberry, wheat, lettuce, and tomato [8,9,10,11,12]. Moreover, other studies have documented the biocontrol capabilities of *B. velezensis*. For example, *B. velezensis* J17-4, an endophytic bacterium isolated from rice stem tissues, can significantly inhibit *Dickeya zeae* [13]. *B. velezensis* NKMV-3 can effectively control *Streptomyces* spp., which causes early blight in tomatoes, and can potentially be used to prepare biocontrol agents for tomato leaf blight [14]. Zaid DS performed phylogenetic characterization of *B. velezensis* HNA3 via comparative genomic analysis and then proposed the potential application value of this strain in biocontrol from a genomic perspective [15].

The antifungal mechanism of *B. velezensis* has gradually come to light with increasing research on the use of this species in biocontrol [16]. The biocontrol mechanisms of *B. velezensis* include antagonism, bacteriolysis, competition, plant resistance induction, and plant growth promotion, among which antagonism is the most critical mechanism for its biocontrol effects. Antagonism is the ability of strains to effectively inhibit the growth of pathogens through the production of antimicrobial metabolites such as lipopeptides and polyketides, thus preventing and controlling soil-borne diseases. Notably, 10% of the genome of *B. velezensis* consists of biosynthetic gene clusters (BGCs), which encode proteins for the synthesis of antimicrobial compounds [17]. These antimicrobial compounds include surfactin, iturin, fengycin, and bacillomycin, which exhibit high antagonistic activity against a wide range of pathogenic microorganisms [18]. In addition to its antagonistic effects, *B. velezensis* can disrupt the cellular structure of pathogens through lysis to achieve inhibition, and a variety of carbohydrate-active enzymes (CAZymes) play a vital role in this process [19]. Progress in sequencing technology development has facilitated ongoing genomic analysis of *B. velezensis* strains sourced from diverse environments, revealing the biocontrol strategies employed by this strain. Further elucidation of the defense mechanism of this strain will be conducive to the rapid development of biocontrol strategies.

*Bacillus* sp. BRI3, which was isolated from soil in this study, was determined to belong to *B. velezensis* via morphological, biochemical, and genome annotation analyses. The antagonistic ability of BRI3 was determined against seven phytopathogenic fungi, such as *Rhizoctonia solani* and *S. sclerotiorum*. BR13 exhibited broad-spectrum inhibitory activity against plant pathogenic fungi, particularly exhibiting greater efficacy against *S. sclerotiorum* compared to the biocontrol type strain FZB42. The antifungal mechanism of BRI3 was investigated through sequencing and annotation of its whole genome. Comparative genomic analysis of BRI3 and the type strain *B. velezensis* FZB42 revealed the abundance of genetic resources in BRI3, with 13 BGCs identified within its genome. Overall, the results of this study demonstrated that BRI3 is a biocontrol strain with potential broad-spectrum antagonism against phytopathogenic fungi and efficient antagonism against *S. sclerotiorum*. This study is anticipated to contribute to the management of *S. sclerotiorum*-related plant diseases by identifying potential strains with significant agricultural value.

## 2. Materials and Methods

### 2.1. Strain Isolation

The soil sample was collected from the experimental field in Dongying City, Shandong Province, China. The soil sample was dissolved in NaCl solution (0.85% *w*/*v*) and subjected to vigorous shaking for 2 min. The soil mixture was subsequently diluted 1000-fold, and 0.1 mL of liquid was uniformly coated on *Bacillus* agar medium (10.0 g/L glucose, 5.0 g/L Ca_3_(PO_4_)_2_, 0.5 g/L (NH_4_)_2_SO_4_, 0.2 g/L KCl, 0.1 g/L MgSO_4_·7H_2_O, 0.0001 g/L MnSO_4_, 0.0001 g/L FeSO_4_, 0.5 g/L yeast extract, and 15.0 g/L agar; neutral pH) and incubated at 37 °C for 12 h. Colonies with a morphology similar to that of *Bacillus* were selected at the end of the culture. The isolates exhibiting the desired characteristics were streaked onto *Bacillus* medium for purification through three successive passages, and the resulting pure colony was designated with the identifier BRI3.

### 2.2. Strains and Culture Media

The strains used in this study included *B. velezensis* BRI3 (isolated and obtained from this study, GDMCC NO. 63555), *B. velezensis* FZB42 (DSM23117), *S*. *sclerotiorum*, *R. solani*, *Fusarium oxysporum*, *Fusarium verticillioides*, *Phytophthora capsici* Leonian, *Corynespora cassiicola*, and *Diplocarpon mali* (further details about the pathogenic fungi are presented in Table S4). *B. velezensis* BRI3 and *B. velezensis* FZB42 were cultured in Lysogenic Broth (LB) medium (5.0 g/L yeast extract, 10.0 g/L peptone, and 10.0 g/L NaCl; neutral pH). The phytopathogenic fungi were cultured on potato dextrose agar (PDA) medium (200.0 g/L potato, 20.0 g/L glucose, and 15.0 g/L agar; neutral pH).

### 2.3. Morphological Analysis

The colony characteristics of BRI3 were recorded after incubation on LB agar plates at 37 °C for 12 h. Gram staining of BRI3 was performed using a Gram staining kit (Solarbio Technology Co., Ltd., Beijing, China).

### 2.4. 16S rRNA Sequencing and Phylogenetic Analysis

BRI3 genomic DNA was extracted according to the instructions of the Omega Bacterial Gene Extraction Kit (Norcross, GA, USA). The obtained genomic DNA was used as a template to amplify 16S rRNA via the forward primer 27F (5-AGAGTTTGATCCTGGCTCAG-3′) and reverse primer 1492R (5′-ACGGCTACCTTGTTACGACTT-3′) [20], which were synthesized by the BGI Tech Solution Beijing liuhe Co., Ltd. (Beijing, China). The PCR products were sequenced by Sangon Biotech (Shanghai) Co., Ltd. (Shanghai, China). Homologous sequences were retrieved and downloaded from the NCBI database (https://www.ncbi.nlm.nih.gov/, accessed on 17 May 2023) and then compared using Clustal X. MEGA 7.0 software was used to construct a 16S rRNA phylogenetic tree using the neighbor-joining method.

### 2.5. Antifungal Activity

Liquid cultures of BRI3 and FZB42 were centrifuged at 4 °C and 2500× *g* for 10 min to remove the supernatant, and the bacterial precipitate was washed with sterile water; this was repeated twice. The washed bacterial pellet was resuspended to an OD_600_ of 1.0. The mycelia of the phytopathogenic fungi were obtained by using a puncher to extract a mycelial plug. A sterilized toothpick was then used to transfer the mycelial plug, with the mycelia facing downward, onto the center of a new PDA plate. At a distance of 2.0 cm from the left and right of the mycelial plug, 10 μL of treated bacterial suspension was added. Sterile water was used as the control. Fungal straight-growth diameters were measured using the crisscross method. The inhibition rate, expressed as the percentage of mycelial growth inhibition, was calculated using the following formula: inhibition rate (%) = ((R1 − R2)/R1) × 100%, where R1 and R2 represent the mycelial growth radius of the pathogen in the control group without BRI3 inoculation and the treatment group with BRI3 inoculation, respectively [21]. The inhibition rate was the mean of three replicates with standard error and was statistically compared via one-way analysis of variance (ANOVA).

### 2.6. Genome Sequencing and Assembly

The genome of BRI3 was sequenced by Beijing Novozymes Technology Co. (Beijing, China). Genomic DNA was extracted using the SDS method, followed by agarose gel electrophoresis to determine the purity and integrity of the DNA, which was quantified using a Qubit (Thermo Fisher Scientific, Waltham, MA, USA). A 10K SMRTbell library was constructed using the SMRT BellTM Template Kit (version 1.0) (Pacific Biosciences of California, Inc., Menlo Park, CA, USA). The DNA samples qualified by electrophoresis were fragmented by a Covaris g-TUBE (Covaris, Inc., Woburn, MA, USA) into target fragments of the size required to construct libraries. Following DNA damage repair and end-repair, hairpin-type junctions were attached to the ends of the DNA fragments via DNA ligases, and the DNA fragments were purified via AMpure PB magnetic beads (Pacific Biosciences of California, Inc., Menlo Park, CA, USA). DNA fragments of a specific size range were selectively isolated using the BluePippin size selection method. The SMRTbell libraries were subsequently subjected to concentration screening using AMpure PB magnetic beads, followed by repair of the damaged DNA. The SMRTBell libraries were subsequently purified again using AMpure PB magnetic beads, and the concentrations of the constructed libraries were quantified by a Qubit. The size of the inserted fragments was determined using an Agilent 2100 system (Agilent Technologies, Inc., Santa Clara, CA, USA), and sequencing was subsequently performed on the PacBio platform. DNA samples that passed the electrophoresis test were randomly cleaved into fragments approximately 350 bp in length using a Covaris ultrasonic crusher. The processed DNA fragments were used with the NEBNext^®^ Ultra™ DNA Library Prep Kit for Illumina (New England Biolabs, Ipswich, MA, USA) to complete library preparation by end repair, the addition of A-tails, the addition of sequencing junctions, purification, PCR amplification, and other steps. After the library was constructed, a Qubit 2.0 was used for preliminary quantification, and the library was diluted to 2 ng/µL. The inserted fragments of the library were subsequently detected using an Agilent 2100 system. After the insert size met the requirement, the effective concentration of the library was accurately quantified using the QPCR method to ensure that the quality of the library was suitable. After passing the library inspection, the different libraries were subjected to PacBio Sequel and Illumina NovaSeq PE150 (Illumina, Inc., San Diego, CA, USA) sequencing according to the effective concentration and target downstream data volume. When processing raw downstream data, reads with low sequencing quality were filtered out, and high-quality reads were retained. Genome assembly of the reads was then performed using the SMRT Link v5.0.1 software [22,23].

### 2.7. Genomic Feature Prediction and Annotation

The assembled genomic sequence was subjected to optimization using the Arrow software. This was followed by a process of prediction and annotation. Coding genes were predicted via GeneMarkS analysis [24]. Among the noncoding RNAs, tRNA genes were predicted via tRNAscan-SE [25], rRNA genes were predicted via RNAmmer [26], and sRNA genes were predicted via Rfam database comparative annotation using the CMSearch program (version 1.1rc4) [27,28]. Next, functional annotation of various databases, such as the widely used GO, KEGG, and COG databases, was carried out for the coding gene sequences [29,30,31,32]. The NR database was used to annotate species for classification on the basis of genomic sequences [33]. AntiSMASH (version 2.0.2) was used to predict genomic secondary metabolite synthesis-related BGCs [34]. The assembled genome sequences were visualized and analyzed using Circos [35] in conjunction with the prediction results for the coding genes.

### 2.8. Comparative Genomic Analysis

The type biocontrol strain *B. velezensis* FZB42 [17], which is highly similar to BRI3 and has a publicly reported complete genome, was selected for comparative genomics analyses, with the BRI3 locus as a reference, using the BRIG (version 0.95). BGCs in the two strains were comparatively analyzed using the default settings of antiSMASH v6.0 (https://antismash.secondarymetabolites.org, accessed on 17 May 2023) [36].

## 3. Results

### 3.1. Organism Information

BRI3 is a Gram-positive, rod-shaped, aerobic bacterium. This strain was grown on LB agar plates for 12 h at 37 °C and developed subcircular creamy–white colonies with irregular edges and smooth, moist surfaces (Figure S1). BRI3 shares similar morphological and physiological characteristics with *Bacillus* spp. The minimum information about the genome sequence (MIGS) of BRI3 is provided in Table S1.

### 3.2. Taxonomic Position of BRI3

The phylogenetic tree, constructed based on the 16S rRNA gene of BRI3 (Figure S2), revealed that BRI3 clustered into a branch with *B. velezensis.* These results indicated that BRI3 was most closely related to *B. velezensis*. Despite the prevalent use of Bergey’s Taxonomic Manual and 16S ribosomal DNA (rDNA) sequences for the taxonomic characterization of *Bacillus* species, the high interspecies sequence homology of 16S rDNA often renders the precise identification of isolated *Bacillus* spp. strains challenging, particularly for distinguishing *B. velezensis*, *B. amyloliquefaciens*, and *B. subtilis* [37]. To elucidate the taxonomic position of strain BRI3, its whole genome was sequenced, and the resulting data were annotated against the NR database to facilitate comprehensive comparative analysis. Whole-genome sequencing of strain BRI3 was performed through services rendered by Novogene. The relatively comprehensive nonredundant protein database (NR) [33] was established and is maintained by NCBI. Furthermore, data pertinent to species classification are also included in the annotation results in the NR database. The species and number of genes annotated with BRI3 in the NR database are shown in Figure S3. The results revealed that among the 4301 genes annotated in strain BRI3 against various species within the NR database, 2035 were affiliated with *B. velezensis*, accounting for 47.31% of the total annotations and constituting the highest proportion. Overall, the annotation results from the NR database were consistent with the 16S rDNA phylogenetic tree analysis, thereby supporting the classification of BRI3 as a member of *B. velezensis*.

### 3.3. Fungal Antagonistic Effect of BRI3

**Figure 1:**
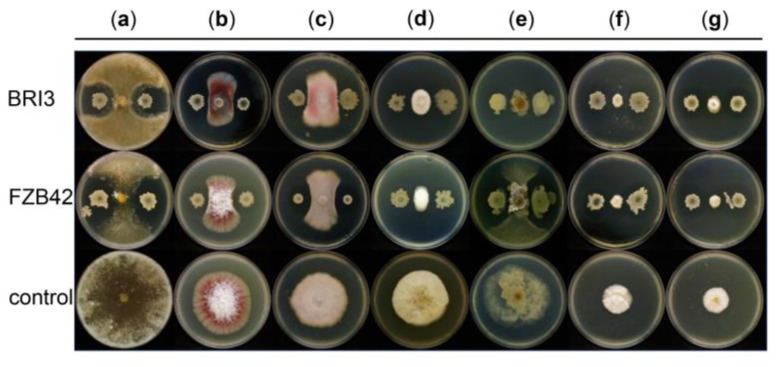
Analysis of the antagonistic activity of *Bacillus velenzensis* BRI3 and *B. velezensis* FZB42 against different fungi on PDA plates. (**a**) *R. solani*; (**b**) *F*. *oxysporum*; (**c**) *F. verticillioides*; (**d**) *P. capsici* Leonian; (**e**) *S. sclerotiorum* (Lib.) de Bary; (**f**) *C. cassiicola*; (**g**) *D. mali*.

*B. velezensis* has been widely studied and applied as a biocontrol strain. To evaluate the inhibitory potential of BRI3 against soil-borne pathogens, the antagonistic activity of BRI3 was examined on the PDA medium. A plate coculture assay indicated that BRI3 may prevent the mycelial growth of diverse pathogens. *R. solani*, *F*. *oxysporum*, *F. verticillioides*, *P. capsici* Leonian, *C. cassiicola*, and *D. mali* were effectively inhibited (>47%) by BRI3 (Figure 1; Table S2), and its inhibitory effect was comparable to that of FZB42. These findings indicate that both BRI3 and FZB42 possess broad-spectrum antimicrobial capacities. Nevertheless, BRI3 strongly inhibits *S. sclerotiorum*, with the inhibition percentage reaching 78.65%, which is significantly greater than that of the type biocontrol strain FZB42. These findings suggest that BRI3 has strong and broad-spectrum inhibitory activity against phytopathogenic fungi.

### 3.4. Genome Features of BRI3

**Figure 2:**
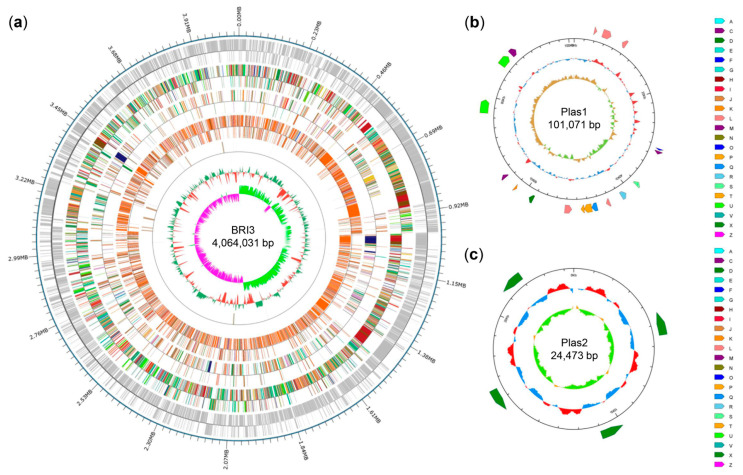
Genome map of the *B. velezensis* BRI3 chromosome. (**a**) Chromosome genome map. The circles, from the outside, represent the following: the first circle represents the coding genes; the second circle represents the annotated results from the COG database; the third circle represents the annotated results from the KEGG database; the fourth circle represents the annotated results from the GO database; and the fifth circle represents the ncRNA distribution. (**b**) Plasmid 1 genome map. (**c**) Plasmid 2 genome map. The circles, from the outside, represent the following: the COG database, genomic sequence position coordinates, and genomic GC content. The annotation information for the letters can be found in Table S3.

To increase our understanding of the high antagonistic potential of the *B. velezensis* strain BRI3 against *S. sclerotiorum*, genomic analysis of this bacterium was conducted. According to the sequencing results, the genome size of *B. velezensis* BRI3 is 4.19 Mb. This genome contains one chromosome (4,064,031 bp) and two plasmids (101,071 bp and 24,473 bp). Figure 2 displays the completed genome assembly and functional annotation, with the outermost circle representing the genomic sequence coordinates. Moving inward, the circles display coding genes, followed by the results of functional annotations derived from the COG, KEGG, and GO databases, and the innermost circle describes noncoding RNAs. A total of 4477 coding sequences (CDSs) were predicted in the BRI3 genome, of which 4307 were predicted for the chromosome, 136 for plasmid 1, and 34 for plasmid 2. The total length of all coding genes was 3.74 Mb, the average length of coding genes was 836 bp, and the total size of the coding region was 89.28% of the whole genome. In addition, a total of 134 noncoding RNAs were identified, consisting of 88 tRNA genes, 18 sRNA genes, nine each of the 16S and 23S rRNA genes, and 10 5S rRNA genes. Furthermore, the analysis revealed 152 tandem repeat sequences comprising 84 TRs and 68 minisatellite DNAs. There were 13 gene islands in the genome, with an average length of 21,230 bp and a total length of 275,993 bp. COG functional classification and a description of the genome map are provided in Table S3.

**Figure 3:**
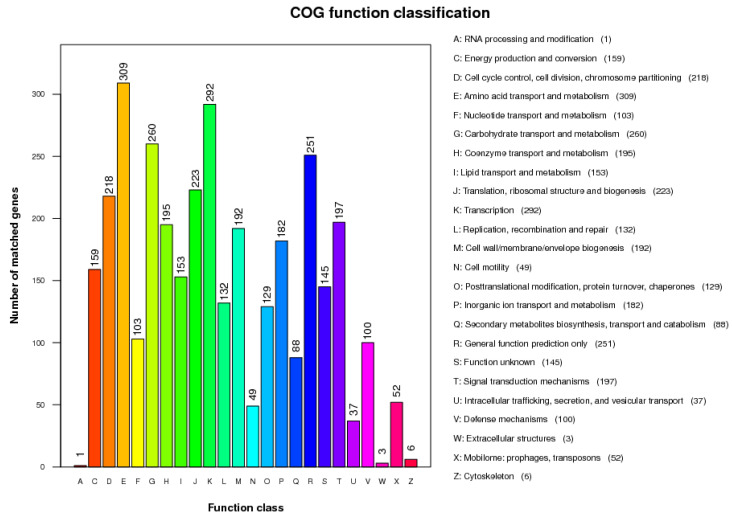
COG categories of proteins encoded in the *B. velezensis* BRI3 genome.

The COG database annotation findings (Figure 3) revealed that the 4477 predicted genes fell into the following categories. There were 829 genes related to genetic information, including in the categories of RNA processing and modification; translation, ribosomal structure, and biogenesis; transcription; replication, recombination, and repair; posttranslational modification, protein turnover, and chaperones; and mobilome: prophages, transposons. The categories of cell cycle control, cell division, chromosome partitioning, cell wall/membrane/envelope biogenesis, cytoskeleton, extracellular structures, intracellular trafficking, secretion, vesicular transport, signal transduction mechanisms, and defense mechanisms were enriched among the 802 genes associated with cells. Additionally, there were 1449 genes related to metabolism, including those related to energy production and conversion; amino acid transport and metabolism; nucleotide transport and metabolism; carbohydrate transport and metabolism; coenzyme transport and metabolism; lipid transport and metabolism; inorganic ion transport and metabolism; and secondary metabolite biosynthesis, transport, and catabolism. Surprisingly, 396 genes with unknown functions or in the general-function-prediction-only category were present in the BRI3 genome, indicating a significant unidentified potential for this strain.

### 3.5. Comparative Genomic Analysis

Given the superior antifungal properties of BRI3 against *S. sclerotiorum* compared with those of the type biocontrol strain FZB42, in this study, a comparative genomic analysis of both BRI3 and FZB42 was performed to elucidate the genetic basis for their differential antifungal capabilities (Table 1). Comparative genomic analysis revealed that the GC content of BRI3 (46.23%) was comparable to that of FZB42 (46.5%). The genome of BRI3 (4,189,575 bp) was 270,979 bp longer than that of FZB42 (3,918,596 bp). Furthermore, BRI3 harbored 4477 CDSs, outnumbering the 3734 CDSs identified in FZB42, suggesting a potential richness in gene information relative to that of its counterpart. Notably, the BRI3 strain contained two plasmids, with lengths of 101,071 bp and 24,473 bp, which were absent in FZB42. This disparity implies that these plasmids might carry genes associated with the enhanced antagonistic activity against *S. sclerotiorum* displayed by the BRI3 strain.

**Figure 4:**
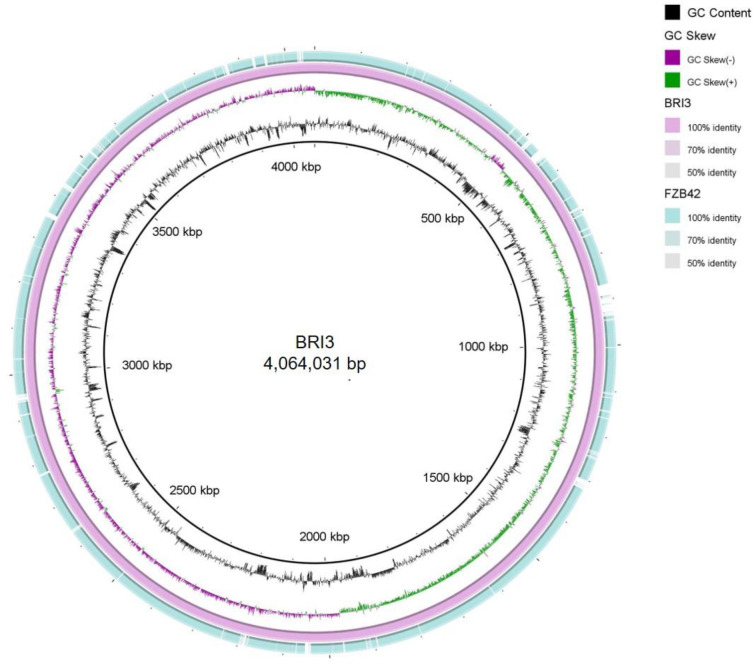
Circular map and genome features of *B. velezensis* BRI3 in comparison with those of other *Bacillus* spp. strains. The circular map was generated using BLAST Ring Image Generator (BRIG) software [38].

Figure 4 depicts a certain degree of chromosomal collinearity between the BRI3 and FZB42 strains; nevertheless, it also reveals the existence of multiple locus-specific gene segments within distinctive regions, such as the intervals from 800 kbp to 1000 kbp, 1200 kbp to 1400 kbp, and 2900 kbp to 3000 kbp, which contribute to the genomic distinctiveness between these two strains. To further evaluate the evolutionary distance between the two strains, the collinearity between the full genomes of BRI3 and FZB42 was assessed using the Mauve program.

In addition, it was crucial to obtain information on the genes contained in the two plasmids in the BRI3 strain. Cluster analysis was carried out based on the COG database annotations of the coding genes of the two plasmids (Table 2). The clustering results revealed that of the 136 coding genes contained in plasmid 1, a total of 20 genes annotated in the COG database could be clustered into the following categories: (1) five genes related to replication, recombination, and repair; (2) three genes related to cell wall/membrane/envelope biogenesis; (3) two genes related to transcription; (4) two genes related to traditional trafficking, secretion, and vesicular transport; (5) one gene related to general function prediction only; (6) one gene related to secondary metabolite biosynthesis, transport, and catabolism; (7) one gene related to signal transduction mechanism; (8) one gene related to post-translational modification, protein turnover, and chaperones; (9) one gene related to cell cycle control, cell division, and chromosome division; (10) one gene related to cell motility; and (11) one gene with unknown function. Among the 34 coding genes contained in plasmid 2, four genes were annotated to the COG database, and all of them were clustered into mobilome: prophages, transposons.

### 3.6. Comparison of Gene Clusters Related to Secondary Metabolites

**Figure 5:**
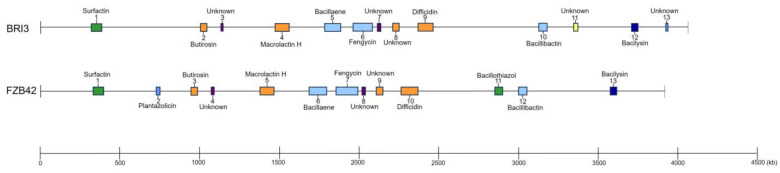
Comparison of the locations and products of secondary metabolite gene clusters in *B. velezensis* BRI3 and *B. velezensis* FZB42.

The antimicrobial activities of *Bacillus* spp. are closely related to their secondary metabolites. A total of 13 BGCs involved in secondary metabolite synthesis were encoded by *B. velezensis* BRI3, according to genome prediction using the antiSMASH-4.0.2 algorithm. These BGCs include three nonribosomal peptide synthetase (NRPS) clusters, three trans-acyl transferase polyketide synthetase (transAT-PKS) clusters, two terpene clusters, one polyketide synthase-like (PKS-like) cluster, one type 3 polyketide synthetase (T3PKS) cluster, one lanthipeptide-class-ii cluster, one other unspecified ribosomally synthesized and post-tRNA-modified peptide (RiPP-like) cluster, and one “other” type of gene cluster. The positions and products of secondary metabolite gene clusters in *B. velezensis* BRI3 and *B. velezensis* FZB42 were compared (Figure 5). Eight clusters (clusters 1, 2, 4, 5, 6, 9, 10, and 12) involved in the biosynthesis of BRI3 core secondary metabolites are also present in strain FZB42. These genes specifically encode surfactin, butirosin, macrolactin H, bacillaene, fengycin, difficidin, bacillibactin, and bacilysin, respectively. Additionally, we discovered five BGCs spanning 118.407 kbp that do not correspond to any previously characterized genes associated with metabolite synthesis. These findings suggest the potential for this strain to generate unexplored secondary metabolites. Notably, among the five uncharacterized BGCs, three presented homology with FZB42, whereas the remaining two (clusters 11 and 13) presented no such homology, suggesting that this strain can synthesize metabolites distinct from those produced by FZB42. The secondary metabolites produced by these two gene clusters might underpin the putative mechanism underlying the strain’s potent antagonistic activity against *S. sclerotiorum*.

**Figure 6:**
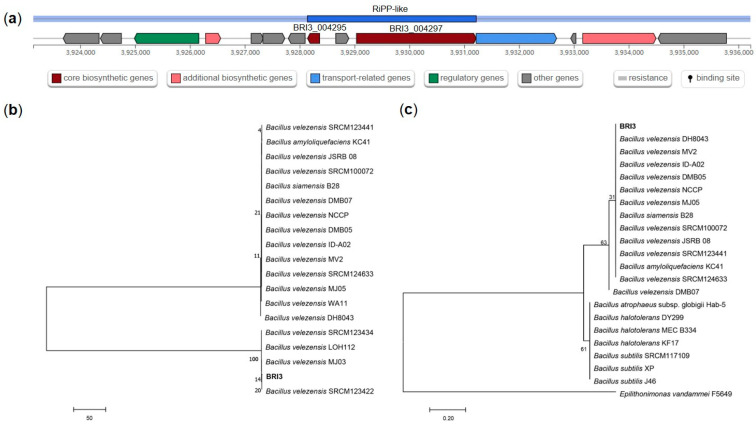
Biosynthetic gene cluster 11 gene structure prediction and phylogenetic analysis. (**a**) Gene structure prediction results. (**b**) Phylogenetic tree of the BRI3_003675 gene. (**c**) Phylogenetic tree of the BRI3_003677 gene.

**Figure 7:**
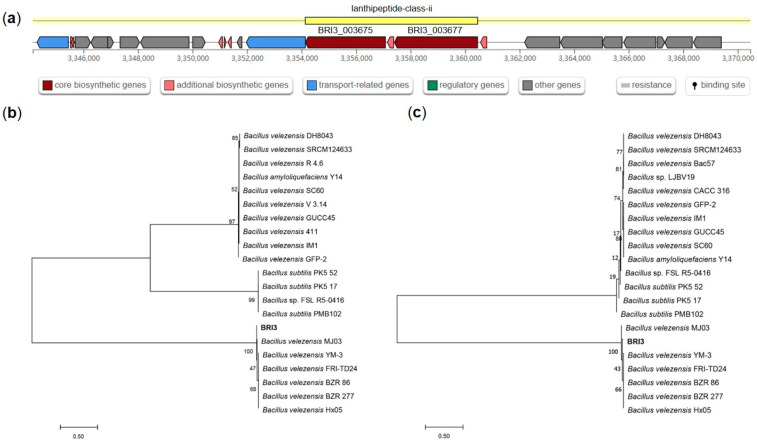
Biosynthetic gene cluster 13 gene structure prediction and phylogenetic analysis. (**a**) Gene structure prediction results. (**b**) Phylogenetic tree of the BRI3_004295 gene. (**c**) Phylogenetic tree of the BRI3_004297 gene.

To further reveal the evolutionary status of these two particular BGCs, we performed homologous sequence comparisons of the predicted core synthetic genes and then compared them using Clustal X. MEGA 7.0 software was used to construct a phylogenetic tree by the neighbor-joining method. According to the antiSMASH prediction (Figure 6), gene cluster 11 contains two core biosynthetic genes (BRI3_003675 and BRI3_003677). The phylogenetic tree revealed that the two core synthetic sequences were most highly homologous in *B. velezensis* and were also found in a few *B. amyloliquefaciens* and *B. subtilis* strains. These findings suggest that cluster 11 represents a suite of genes that are highly conserved in *Bacillus* species, exhibiting a particularly high degree of homology in *B. velezensis* strains. Notably, the type biocontrol strain *B. velezensis* FZB42 lacks the homologous sequence of cluster 11, implying that BRI3 has the potential to synthesize unique biocontrol compounds through this cluster, which could account for its greater efficacy in antagonizing *S. sclerotiorum* than FZB42. Gene cluster 13 also contains two core biosynthetic genes (BRI3_004295 and BRI3_004297) (Figure 7). Phylogenetic analysis revealed that the BRI3_004295 gene was predominantly clustered in the branch with *B. velezensis* and was also found in a few *B. amyloliquefaciens* and *B. subtilis* strains. The BRI3_004297 gene was found in several species of *Bacillus*, exhibiting the highest homology with *B. velezensis*, followed by *B. amyloliquefaciens*, *B. artophaeus*, *B. halotolerans*, and *B. subtilis*. It was also present in *Epilithonimonas vannamei,* but with lower homology. These findings indicate that the cluster 13 biosynthesis-related gene shares the highest homology with *B. velezensis* but is absent in FZB42, suggesting a high level of genetic distinction and underscoring the potential of this gene cluster to produce unique compounds distinct from those identified in the reference strain FZB42.

## 4. Discussion

Numerous diverse rhizospheric microorganisms are found around plant roots and play crucial roles in plant-associated physiological functions such as immune system defense, nutrient uptake, and plant growth [39,40,41]. *B. velezensis* represents a novel species within *Bacillus* spp., and there is ample empirical evidence of its biocontrol efficacy, but strains of this species have different targets and mechanisms of action. As reported in previous studies, *B. velenzensis* BS87 and RK1 exhibit high levels of antagonism toward *F. oxysporum* f. sp. *Fragariae* [42]. The genome of *B. velezensis* B-4 was described by Zhu Z [43], who reported that this strain effectively inhibited the growth of *Thatephorus cucumeris*, *Fusarium graminearum,* and *S. sclerotiorum*. It has been shown that iturin A and surfactin are two key metabolites produced by *B. velezensis* KTA01, significantly contributing to its antagonistic effect against *Botryosphaeria dothidea*. Additionally, the degradation of fungal cell walls by *B. velezensis* KTA01 has been identified as a substantial contributing factor to its biocontrol efficacy [44]. The type strain *B. velezensis* FZB42 has been extensively employed in the field of biological control, harboring a genome enriched with genes responsible for the synthesis of various antibiotics and bacteriocins, such as polyketides and lipopeptides. The mechanisms underlying the antimicrobial activity associated with these compounds have been the subject of extensive investigations [17,45,46,47,48,49,50,51]. In this study, *B. velezensis* BRI3, which was isolated from rhizosphere soil, exhibited broad-spectrum antifungal activity against plant pathogenic fungi. This characteristic was comparable to that of the type strain FZB42 in terms of effectiveness. Specifically, BRI3 displayed a particularly potent inhibitory effect on *S. sclerotiorum*, outperforming the type strain FZB42. *S. sclerotiorum* is a known pathogen that affects crops and vegetables, causing diseases in monocotyledonous and dicotyledonous plants such as oilseed rape and sunflower. Biocontrol studies of *S. sclerotiorum* have reported that volatiles produced by *B. endophytes* can effectively inhibit the growth of *S. sclerotiorum* [52]. Additionally, *Coniothyrium minitans* has been shown to inhibit *S. sclerotiorum* expansion through the activity of fungal cell wall-degrading enzymes (FCWDs) [53]. Traditional chemical pesticides have long been employed effectively for plant disease control. However, concerns over their potential risks to food safety and environmental pollution are increasing, necessitating a shift toward more sustainable and environmentally friendly alternatives. Biological control methods have emerged as vital strategies and represent a future trend in plant disease management. Therefore, the discovery of the BRI3 strain and the genomic insights unveiled in this research significantly contribute to the management and understanding of *S. sclerotiorum*, thereby enhancing our ability to combat this pathogen.

*Bacillus* spp. can secrete many secondary metabolites that have strong biological activities, such as antimicrobial, antiviral, and nematocidal activities [54,55,56]. The antibiotic secondary metabolites produced by *Bacillus* spp. predominantly include nonribosomal peptides (NRPs), polyketides, and their associated derivative compound, which exhibit favorable antimicrobial properties and surfactant activity, indicating potential applications in the realms of biopharmaceuticals, agricultural production, and environmental management [57]. Surfactin and fengycin belong to the group of NRPs that have been reported as essential antagonists of phytopathogenic fungi, but their antifungal mechanisms have not been fully elucidated. Li et al. revealed that surfactin and fengycin contained in the bacterial culture supernatant of *B. subtilis* GLB19 can effectively protect grapes from *Botrytis cinerea* [58]. Polyketides constitute the other dominant family of secondary metabolites with relevant bioactivities. Macrolactin H, a polyketide antibiotic generated by polyketide synthase (PKS), has potentially promising applications in aquaculture, exhibiting antagonistic effects against *Aeromonas* spp. and *Vibrio* spp. [59]. Bacillaene is a linear molecular compound with two amide bonds synthesized by PKS [60]. Patel PS et al. empirically demonstrated through in vitro studies that this antibiotic exerts its bacteriostatic effect by inhibiting protein synthesis in prokaryotic cells [61]. Difficidin, encoded by cluster 9 of the secondary metabolite synthesis genes of BRI3, is a type of polyketide with broad-spectrum antimicrobial activity [62]. Chen XH et al. reported that bacilysin and difficidin produced by *B. amyloliquefaciens* FZB42 (now categorized as *B. velezensis* FZB42) were effective in preventing and controlling fire blight in fruit trees. In addition, the bioactivity of secondary metabolites from other classes has also been confirmed. The siderophore bacillibactin produced by *B. amyloliquefaciens* MTCC 12713 potentially inhibits methicillin-resistant *Staphylococcus aureus*, vancomycin-resistant *Enterococcus faecalis*, *Pseudomonas aeruginosa,* and *Klebsiella pneumoniae* [63]. Butirosin is an amino acid glycoside with inhibitory activity against some Gram-positive and Gram-negative bacteria [64]. Amidst the escalating environmental threats posed by conventional chemical pesticides, interest in the utilization of biopesticides has increased [65]. The application of biopesticides in horticulture, agriculture, and livestock has indeed steadily increased, as noted by Glare et al. [66]. However, biopesticides continue to face significant challenges [67], which include short shelf life, the immaturity of formulation improvement technology, the registration of products, etc. Comparative genomics analysis revealed that BRI3 encodes 13 BGCs, eight of which are homologous to those found in FZB42, synthesizing a range of bioactive compounds, including Surfactin, Fengycin, Macrolactin H, Bacillaene, Difficidin, Bacilysin, Bacillibactin, and Butirosin, as previously delineated. In addition to the eight reported BGCs homologous to FZB42 mentioned above, the BRI3 genome contains five uncharacterized BGCs as well as two uncharacterized BGCs that are not homologous to FZB42, suggesting that new antimicrobial compounds may be produced by BRI3. To explore this novelty, we conducted a phylogenetic analysis by constructing a homology-based phylogenetic tree using the core biosynthetic sequences from these two distinct BGCs. The results revealed high homology among the sequences within *B. velezensis*. Unexpectedly, the type biocontrol strain FZB42, which is also classified as a *B. velezensis* strain, lacks the aforementioned pair of BGCs. This observation suggests that the presence of these two BGCs is not a universal characteristic among biocontrol strains, suggesting that the compounds synthesized by these BGCs could account for the strain’s potent antifungal activity against *S. sclerotiorum*.

In addition, the genome of the type strain FZB42 contains no plasmids and only one chromosome [68]. The BRI3 genome contains two plasmids carrying approximately 12.5 kbp of genes, suggesting that this strain contains much more genetic information than FZB42. Based on the above findings, it is hypothesized that the high efficacy of *B. velezensis* BRI3 in antagonizing *S. sclerotiorum* is intricately linked to as-yet-uncharacterized BGCs and the genetic information carried by its two plasmids.

In summary, the findings of this study demonstrate that BRI3 is a biocontrol strain with potential broad-spectrum antagonism against plant pathogenic fungi and can effectively antagonize *S. sclerotiorum*. Additionally, this strain can promote the growth of various plants, including maize, rice, and rapeseed; however, the specific results of these observations are not shown in this study. The potential application of this strain in the management of *S. sclerotiorum*-related plant diseases and its application in agricultural production is evident.

## 5. Conclusions

Compared with the biocontrol strain FZB42, *B. velezensis* BRI3, which is isolated from rhizosphere soil, has broad-spectrum antimicrobial activity against seven phytopathogenic fungi and a particularly strong antagonistic effect against *S. sclerotiorum*. This novel isolated strain represents a promising candidate for efficient biocontrol of *S. sclerotiorum*. Whole-genome sequencing and analysis revealed the structure and function of the BRI3 genome. The BRI3 genome consists of one chromosome and two plasmids, harboring a wealth of genetic information. Among the 4477 CDSs, 24 types of gene clusters were identified, encompassing secondary metabolism, transport, catabolic pathways, etc. A total of 13 BGCs were predicted in the BRI3 genome, highlighting two unique gene clusters in BRI3 that could contribute to the biosynthesis of unique molecules. In conclusion, our study provides genomic insights into BRI3 and highlights its potential as a valuable resource for agricultural applications, particularly in the management of *S. sclerotiorum*. Fungal-related plant diseases can be effectively controlled through biocontrol. Further research is indispensable for harnessing the full potential of these genomic traits to develop pragmatic strategies for disease control.

## Figures and Tables

**Table 1:** Genomic features of *B. velezensis* BRI3 and comparison with those of *B. velezensis* FZB42.

	FZB42	BRI3		
Genome size (bp)	3,918,596	4,189,575	Chr1	4,064,031
			Plas1	101,071
			Plas2	24,473
Total GC content (%)	46.5	46.23		
Total genes	3855	4611		
Total protein-coding genes (CDS)	3734	4477		
Total rRNA genes	29	28		
Total tRNA genes	88	88		
Number of plasmids	0	2		
Isolation source	infested sugar beet	soil		
GenBank sequence	NC_009725.2	CP171807.1		

**Table 2:** COG categories encoding proteins in the two plasmids.

Plasmid	COG	Number
Plas1	Replication, recombination, and repair	5
	Cell wall/membrane/envelope biogenesis	3
	Transcription	2
	Intracellular trafficking, secretion, and vesicular transport	2
	General function prediction only	1
	Secondary metabolite biosynthesis, transport, and catabolism	1
	Signal transduction mechanisms	1
	Post-translational modification, protein turnover, and chaperones	1
	Cell cycle control, cell division, and chromosome partitioning	1
	Cell motility	1
	Function unknown	1
Plas2	Mobilome: prophages, transposons	4

## Data Availability

The data presented in this study are openly available in NCBI, accession number CP171807.1.

## Supplementary Materials

The following supporting information can be downloaded at: https://www.mdpi.com/article/10.3390/genes15121588/s1, Figure S1: Morphological characteristics of *B. velezensis* BRI3. (**a**) Morphological characteristics of BRI3 on LB plate. (**b**) Image of single colony morphology. (**c**) Gram-positive staining of BRI3; Figure S2: Phylogenetic tree of BRI3 with closely related species drawn by neighbor-joining method based on 16S rRNA gene sequences. Bootstrap values (%) based on 1000 replications were given at nodes. Bar, 0.01 substitutions per nucleotide position; Figure S3: Species and the number of genes with the BRI3 genome annotated in the NR database; Table S1: Minimum information about the genome sequence of *B. velezensis* BRI3; Table S2: Antagonistic efficiency of *B. velezensis* BRI3 against pathogenic fungi; Table S3: COG functional classification and description; Table S4: The phytopathogenic fungi used in this study.
