# Supplementary material for: Comparative Genomic Analysis of Bacillus velezensis BRI3 Reveals Genes Potentially Associated with Efficient Antagonism of Sclerotinia sclerotiorum (Lib.) de Bary
Source: Genes (Basel). 2024 Dec 11;15(12):1588. doi: 10.3390/genes15121588 (PMC11675273; doi:10.3390/genes15121588)
Supplement: Supplementary file 1 [file genes-15-01588-s001.zip › genes-3338698-supplementary.pdf]

Supplementary Information

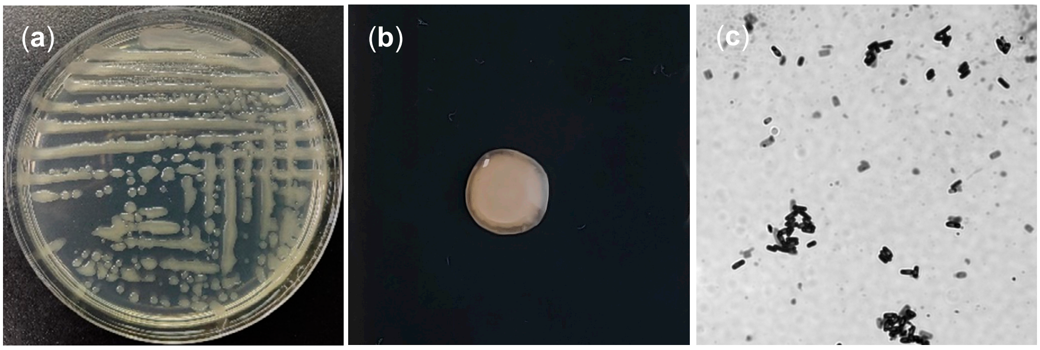

Figure S1. The morphological characteristics of *Bacillus velezensis* BRI3. (**a**) The Morphological characteristics of BRI3 cultured on LB agar plate for 12 hours at 37°C. (**b**) Image of single colony morphology. (**c**) Gram-positive staining of BRI3.

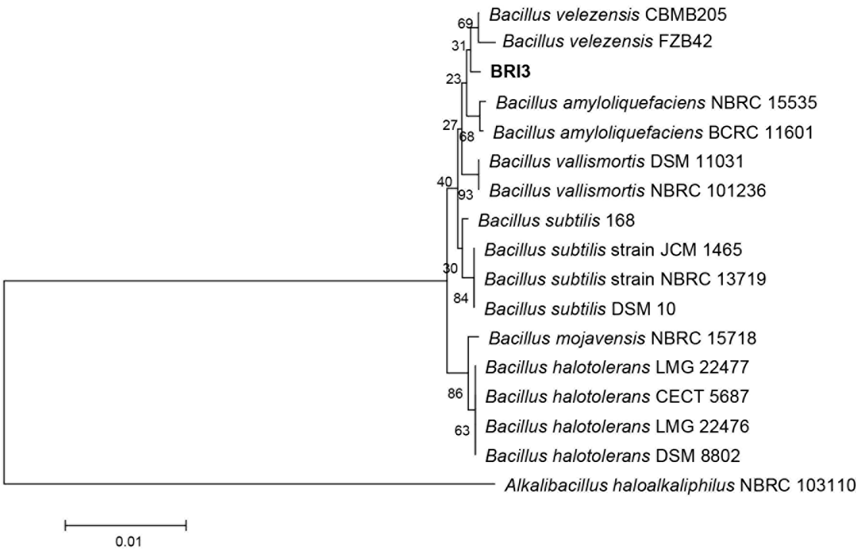

Figure S2. Phylogenetic tree of BRI3 with closely related species drawn by Neighbor-Joining method based on 16S rRNA gene sequences. Bootstrap values (%) based on 1000 replications were given at nodes. Bar, 0.01 substitutions per nucleotide position.

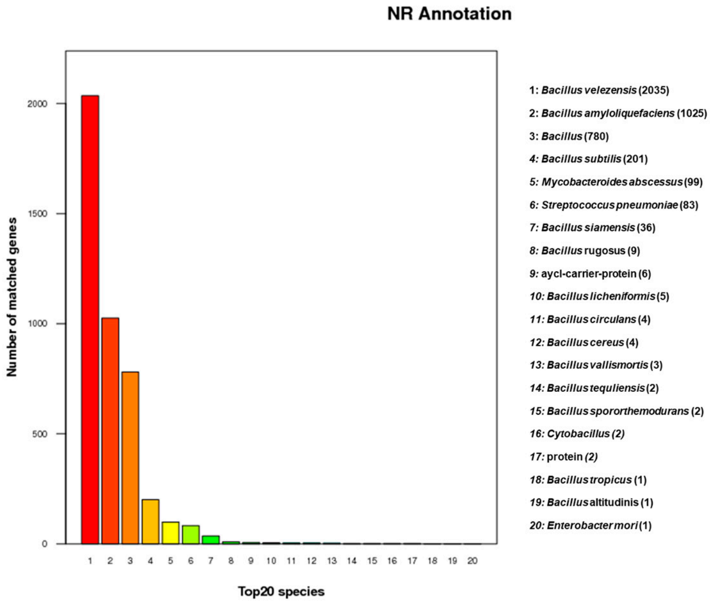

Figure S3. Species and the number of genes with the BRI3 genome annotated in the NR database
*The protein sequences of the predicted genes were subjected to Diamond comparison with the NR database (evalue ⩽ 1e-5). The comparison result with the highest score (default identity ⩾ 40%, coveraged ⩾ 40%) was selected for annotation.

Table S1. Minimum information about the genome sequence of *Bacillus velezensis* BRI3

Item	Description
Project name	*B. velezensis* BRI3
Organism	Bacteria; Firmicutes; *Bacilli*; *Bacillales*; *Bacillaceae*; *Bacillus*; *B. velezensis* BRI3
Isolate type	Monoisolate
Sample type	Chromosomal DNA; Plasmidic DNA
Soil environmental package	Soil samples of agricultural field
Sequencing platforms	PacBio; Illumina PE150
Number of Reads	177933
Number of Bases	1977684760
Mean Read Length	11115
Assembly method	SMRT Link
Polished Contigs	7
Max Contig Length	4091904
Sum of Contig Lengths	4363107

*Polished Contigs: Number of contigs assembled; Max Contig Length: Maximum contig length in the sequence obtained by assembly.

Table S2. Antagonistic efficiency of *Bacillus velezensis* BRI3 and FZB42 against pathogenic fungi

**Pathogen**	**Antagonistic efficiency（%）**	
	**BRI3**	**FZB42**
Rhizoctonia solani	76.89±1.11	79.17±0.83
Fusarium oxysporum	60.40±2.01	60.12±3.04
Fusarium verticillioide	54.45±2.07	62.50±3.57
Phytophthora capsici Leonian	57.21±1.44	56.67±6.67
Sclerotinia sclerotiorum (Lib.) de Bary	78.54±3.54*	72.22±3.89
Corynespora Cassiicola	54.45±2.07	54.17±2.23
*Diplocarpon mali Y.*Harada et Sawamura	47.49±11.13	45.93±1.05

*Fungal straight growth diameters were measure via the crisscross method.

*FZB42 is the representative type biocontrol strain.

*Values were the mean of three replicates with standard error and analyzed by one-way analysis of variance (ANOVA). *, a significant difference between the BRI3 group and FZB42 group (p<0.05). The ANOVA assumptions were confirmed by Brown-Forsythe test.

Table S3. COG Functional classification and description

Functional class	Class description
A	RNA processing and modification
C	Energy production and conversion
D	Cell cycle control, cell division, chromosome partitioning
E	Amino acid transport and metabolism
F	Nucleotide transport and metabolism
G	Carbohydrate transport and metabolism
H	Coenzyme transport and metabolism
I	Lipid transport and metabolism
J	Translation, ribosomal structure and biogenesis
K	Transcription
L	Replication, recombination and repair
M	Cell wall/membrane/envelope biogenesis
N	Cell motility
O	Posttranslational modification, protein turnover, chaperones
P	Inorganic ion transport and metabolism
Q	Secondary metabolites biosynthesis, transport and catabolism
R	General function prediction only
S	Function unknown
T	Signal transduction mechanisms
U	Intracellular trafficking, secretion, and vesicular transport
V	Defense mechanisms
W	Extracellular structures
X	Mobilome: prophages, transposons
Z	Cytoskeleton

Table S4 The phytopathogenic fungi used in this study

Phytopathogenic fungi	Accession number
Rhizoctonia solani	ACCC 36441
Fusarium oxysporum	ACCC 39252
Fusarium verticillioides	ACCC 39254
Phytophthora capsici Leonian	ACCC 37284
Sclerotinia sclerotiorum (Lib.) de Bary	ACCC 37707
Corynespora Cassiicola	ACCC 37618
Diplocarpon mali	ATCC 64308
